# An Attention-Enhanced Multi-Scale and Dual Sign Language Recognition Network Based on a Graph Convolution Network

**DOI:** 10.3390/s21041120

**Published:** 2021-02-05

**Authors:** Lu Meng, Ronghui Li

**Affiliations:** College of Information Science and Engineering, Northeastern University, Shenyang 110000, China; 1970641@stu.neu.edu.cn

**Keywords:** sign language recognition, GCN, attention mechanism, keyframes extraction, large-vocabulary

## Abstract

Sign language is the most important way of communication for hearing-impaired people. Research on sign language recognition can help normal people understand sign language. We reviewed the classic methods of sign language recognition, and the recognition accuracy is not high enough because of redundant information, human finger occlusion, motion blurring, the diversified signing styles of different people, and so on. To overcome these shortcomings, we propose a multi-scale and dual sign language recognition Network (SLR-Net) based on a graph convolutional network (GCN). The original input data was RGB videos. We first extracted the skeleton data from them and then used the skeleton data for sign language recognition. SLR-Net is mainly composed of three sub-modules: multi-scale attention network (MSA), multi-scale spatiotemporal attention network (MSSTA) and attention enhanced temporal convolution network (ATCN). MSA allows the GCN to learn the dependencies between long-distance vertices; MSSTA can directly learn the spatiotemporal features; ATCN allows the GCN network to better learn the long temporal dependencies. The three different attention mechanisms, multi-scale attention mechanism, spatiotemporal attention mechanism, and temporal attention mechanism, are proposed to further improve the robustness and accuracy. Besides, a keyframe extraction algorithm is proposed, which can greatly improve efficiency by sacrificing a little accuracy. Experimental results showed that our method can reach 98.08% accuracy rate in the CSL-500 dataset with a 500-word vocabulary. Even on the challenging dataset DEVISIGN-L with a 2000-word vocabulary, it also reached a 64.57% accuracy rate, outperforming other state-of-the-art sign language recognition methods.

## 1. Introduction

Sign language is the most important way of communication between hearing-impaired people. It plays an irreplaceable role in the hearing-impaired community, but most normal people cannot understand it. Therefore, research on automatic sign language recognition algorithms will help build a bridge of communication between hearing-impaired people and others, which will greatly facilitate the life of hearing-impaired people. Sign language mainly conveys semantic information through hand shapes, motion trajectory, facial expressions, lip movements, and eye contact, etc. It is usually composed of one or more gestures, movements, and transitions between them. A slight change in one of these components may lead to another completely different meaning.

According to different data modalities, sign language recognition can be divided into sensor-based and vision-based methods. Some researchers use sensors such as EMU, data gloves or IMUs to recognize sign language. Zhang et al. [1] combined 3-axis accelerometer signals and the 5-channel EMG signal on the user’s hand to recognize 72 sign language words with 93.1% accuracy. The sensor-based methods have fast recognition speed and high accuracy, but it is inconvenient for signers to wear sensors for the following reasons: (1) the users have to take the electronic devices with them, which could be a burden for people; (2) all the portable electronic devices need batteries that have to be charged frequently; (3) signals from the wearable devices can only be processed by the specific equipment instead of commonly used cameras. In contrast, vision-based methods have the advantages of low cost and convenience, and the users do not have to take anything with them, just need to “say” the words in front of a common cameras, and the others can understand what they’re expressing. For example, setting up vision-based sign language translators at ticket counters and bank counters could greatly facilitate the daily life of hearing-impaired people. Therefore, vision-based methods have become the main research direction of sign language recognition. However, there are still several problems in vision-based sign language recognition:

- low recognition efficiency caused by too much redundant information.
- poor recognition accuracy caused by finger occlusion and motion blurring.
- poor generalization of algorithms caused by differences in signing style between sign language speakers.
- small recognizable vocabulary caused by the existence of similar words in large vocabulary datasets.

In this work, we propose an attention-enhanced multi-scale and dual Sign Language Recognition Network based on Graph Convolution Network (GCN), which is capable if matching the performance of the state-of-the-art on two large Chinese sign language datasets. A large body of work has been proposed for sign language recognition (SLR) [2,3]. Before 2016, the traditional sign language recognition technology based on vision has been studied extensively, see [4] for details. Traditional sign language recognition methods are complex to implement, and can only recognize limited vocabularies, which cannot fully express human’s intelligent understanding of sign language. In recent years, deep learning technology has greatly exceeded the performance of manual features in many computer vision tasks and therefore has become a new method for sign language recognition.

Many vision-based methods have used video RGB data for sign language vocabulary recognition. Vincent et al. [5] combined a Convolutional Neural Network (CNN) and a Long Short-Term Memory Network (LSTM) for the recognition of American Sign Language words, and used data enhancement techniques such as scaling and smoothing to improve the generalization of the network. Huang et al. [6] proposed a 3D-CNN network based on spatiotemporal attention mechanism for large vocabulary sign language recognition.

Some research works have used depth images, skeleton data, optical flow, and other different modal data for identification. Duan et al. [7] combined RGB data, depth images, and optical flow to recognize isolated gestures. They provided a convolutional two-stream consensus voting network (2SCVN) to explicitly model the short-term and long-term structure of the RGB sequences. To reduce the interference of complex backgrounds, a 3d depth-saliency convolution network (3DDSN) is used in parallel to extract motion features. The two networks, 2SCVN and 3DDSN, have been integrated into a framework to improve recognition accuracy. Huang et al. [8] proposed a deep sign language recognition model using a 3D CNN from multi-modal input (including RGB, depth, and skeleton data) to improve recognition accuracy. They verified the model’s effectiveness on their dataset and reported a recognition accuracy of 94.2%.

Recognition algorithms based on multi-modal data can extract various features of different modal data, and while improving accuracy, they also greatly increase the computational complexity. With the development of human pose estimation technology, we can extract the skeleton data of the body and hands from a single RGB frame [9,10]. Compared with other modal sign language data, skeleton data reduces a lot of redundant information and is more robust to lighting and scene changes.

In sign language recognition, there have been two methods to extract skeleton data features in the past. One is to map the skeleton data to the image and use a CNN for processing. For example, Devineau et al. [11] proposed a CNN algorithm based on hand skeleton data for the recognition of three-dimensional dynamic gestures, using parallel convolution to process the position sequence of hand joints, and achieved high recognition accuracy. The other method is to use the Recurrent Neural Network (RNN) to recognize the skeleton data. For instance, Konstantinidis et al. [12] used the multi-stream LSTM algorithm to recognize an Argentine sign language dataset (LSA64). However, neither RNNs nor CNNs can fully represent the structure of skeleton data, because skeleton data is natural graph data, not sequences data or European data. Yan et al. [13] first applied a graph convolution network (GCN) to model skeletal data for the field of action recognition. The model they developed which aims to use body skeleton data to recognize some daily actions like sit up, bowing, etc., is named ST-GCN. After that, various GCN algorithms for action recognition have been proposed. Shi et al. [14] proposed an adaptive algorithm to construct graph data, and alternately used spatial convolution and temporal convolution to learn spatial and temporal features. Liu et al. [15] proposed the MS-G3D network to learn different levels of semantic information by using multiple parallel GCN networks, which inspired us. Si et al. [16] used GCN to learn the spatial features of each frame separately and then used LSTM to learn the temporal features to recognize actions. Although these algorithms have achieved great success in the field of action recognition, there are still many demerits in applying them to the task of sign language recognition:

(1) The graph structure is fixed and is constructed through the natural connection of human bones, which may not be suitable for SLR. For example, in many sign language vocabularies, the relationship between the left and right fingers is significant, but in natural connection, their distances are too long to allow GCN to learn the dependencies between the joints over such long distances. (2) The above methods learned spatial and temporal features separately, so that the complex spatiotemporal features in sign language cannot be learned. (3) Due to the phenomenon of figure occlusion, some hand joints are difficult to accurately identify, and these occluded joints are not so important for recognizing this word. If they are treated equally, this can easily cause misjudgments. (4) The temporal dependences of sign language are longer than that of actions. This includes sign language actions and transition actions. The former are the key to recognition, while transition actions are interference. The above algorithms treat sign language movements and transition movements equally. Moreover, there are motion blur frames in sign language videos, which makes it difficult to accurately extract the joint points of this frame, which seriously affects the subsequent recognition accuracy.

To address these shortcomings, we proposed our method based on the following hypotheses: (1) a human’s head, arms and hands can clearly express the sign language information, which can be analyzed and processed using mathematical graph theory; (2) it is better to use spatiotemporal features from the video frames than spatial or temporal features separately; (3) although there are a lot of frames in the sign language video, we believe that not all the frames play the same roles, and attention mechanism and key frames technique can improve the accuracy and speed of the algorithm.

In this work, we first extracted the skeleton data of the body, hands, and part of the face from the RGB images based on the works of [9,10]. The original skeleton data is normalized to eliminate the differences in height and body shape of different sign language speakers. We applied the GCN algorithm to sign language recognition tasks for the first time and proposed a multi-scale attention network (MSA) to learn the long-distance dependencies, which can model the dependencies between remote vertices without considering the distance. We also proposed a multi-scale spatiotemporal attention network (MSSTA) to learn the complex spatiotemporal dependencies in sign language. Aiming at the problem of long-temporal dependencies in sign language and inaccurate recognition of motion blur frame joints, we proposed an attention enhanced temporal convolutional network, which can automatically assign different weights to different frames, thereby improving the recognition accuracy. For example, blurry motion frames are often not helpful for vocabulary recognition, so under the action of temporal attention, the weight of the blurred frame should be minimal, thereby improving the robustness of the algorithm. Finally, two-stream network integration of joints and bone data is used to improve performance. Besides, we also proposed a keyframe detection algorithm, which can significantly improve the practice of the algorithm while maintaining high recognition accuracy.

In summary, the main contributions of our work are: (1) Estimate the skeleton data of the body, hands, and part of the face from the RGB data, and mormalize the original skeleton data to eliminate the differences in height and body shape caused by different sign speakers. (2) We used the GCN algorithm to isolate sign language recognition for the first time, which provides a new idea for sign language recognition. We designed the SLR-Net network, which allows the GCN network to directly learn the spatiotemporal features and the dependencies between long-distance vertices. (3) We proposed three attention mechanisms based on SLR-Net to further improve the robustness and accuracy of the algorithm. (4) We proposed a keyframe extraction algorithm, which can greatly improve recognition efficiency while maintaining high recognition accuracy. (5) We conducted a lot of experiments on two large-vocabulary public sign language datasets and reached state of the art.

## 2. Related Work

### 2.1. Sign Language Recognition Based on CNN

Due to the basic status of convolutional neural networks (CNN) in deep learning networks, some research teams have conducted a series of CNN-based isolated sign language recognition studies since 2013 [6,17,18,19,20,21,22,23,24,25]. Based on CNN recognition, the algorithm can be optimized by adding multi-modal data (including depth, skeleton, key points of the human body, etc.), detecting hand regions, and feature fusion. Literature [26,27,28,29] proposed a sign language recognition CNN network based on multi-modal data, which can use multi-scale to capture image features at various levels. Kopuklu et al. [28] proposed a data-level fusion strategy for fusing motion information into static images, and sent the fused spatiotemporal features to the CNN network for subsequent classification, and achieved commendable recognition.

The hands’ area is undoubtedly the most important areas in sign language recognition. Therefore, some research works use detection or tracking algorithms to extract the hand’s areas. Kim et al. [30,31] used the target detection network to find the hands’ area and combined the original sign language data to feed the CNN network, which improved the accuracy and reduced the training time by half. Although traditional 2D CNN has strong feature extraction capabilities, it is not very suitable for the input of multi-frame image data. Sign language recognition also needs to adopt some methods that can extract the correlation between frames, so researchers used 3D Convolutional Neural Network (3DCNN) to achieve more global recognition, which can learn spatiotemporal features and capture motion information. Liang et al. [32] proposed a sign language recognition algorithm based on multi-modal data and 3D-CNN network and verified its effectiveness on a large-scale dataset.

Some researchers map skeleton data into pseudo-images and use CNN algorithms for recognition. Kumar et al. [33] identified the 3D joint coordinates of the human body and hands from the RGB image, then encoding joint angular displacement maps (JADMs) into color texture images for use in CNN-based recognition.

### 2.2. Sign Language Recognition Based on RNN

Compared with the network architecture described above, a Recurrent Neural Network (RNN) is a type of network suitable for processing sequence data, which is better at capturing long-term contextual semantic information. Therefore, in recent years, many works on sign language recognition based on RNN have emerged.

Huang et al. [34] proposed an RNN-based sign language recognition algorithm, which embedded the keyframe algorithm into the RNN network, which allowed different levels of attention to the input frame and achieved remarkable recognition results. Liao et al. [35] proposed a sign language recognition framework based on the BLSTM network in 2019. First, they use the detection network to extract the hand region, then input the hand region and the original RGB data into LSTM, to achieve dynamic long-temporal sequence feature modeling, and finally output the classification results. Yang et al. [36] proposed a method of combining CNN with LSTM, where they used RGB and optical flow data as two inputs and fused them at the full connection layer to output classification results. It is evaluated on the constructed small-scale sign language dataset they constructed and meets the real-time requirements of a small-scale sign language recognition system. Konstantinidis et al. [12] used LSTM to model skeleton data to recognize sign language words and finally used 8-stream network fusion in the softmax layer to improve accuracy.

### 2.3. Graph Convolution Network for Action Recognition

In sign language recognition, the skeleton data of the body and hands are sufficient to represent most sign language words. Some works used a method like CNN or RNN to model skeleton data, which is inefficient. Skeleton data is natural graph data, which is very suitable for modeling with GCN algorithm. Gori et al. [37] first proposed the graph neural network (GNN), which is more suitable for dealing with non-Euclidean data. The vertices of the graph represent the object information, and the edges represent the relationship between the nodes.

Inspired by the great success of the convolution network, Bruna et al. [38,39,40] created the GCN algorithm, which extends the graph data to the frequency domain for convolution operation. Yan et al. [13] designed the ST-GCN network, first applied the GCN algorithm in the field of action recognition, and achieved great success on the NTU dataset and the kinects dataset, providing a new direction for the field of action recognition. Shi et al. [14] designed the 2S-AGCN algorithm based on the work of [13]. The main contribution is the design of an adaptive graph convolution algorithm and the fusion of two-stream GCN. Plizzari et al. [41] used the idea of word embedding algorithms to remap the input skeleton data. They designed a spatial attention module SSA and temporal attention module TSA to improve accuracy. Cheng et al. [42] introduced a lightweight shift operation into the GCN algorithm. They proposed a spatial shift operation and a temporal shift operation, which improved GCN’s operating efficiency and can automatically adjust the receptive field. Its performance is better than that of the conventional model.

Although some GCN-based action recognition algorithms have achieved great success, there is still a significant gap in their application to sign language recognition. Due to the inaccurate recognition of hand joints and the longer spatial and temporal dependencies of sign language, it is necessary for the algorithm to have good robustness and extract complex spatiotemporal dependencies.

## 3. Method

### 3.1. Preliminaries

#### 3.1.1. Construction of Graph Data

**Figure 1:**
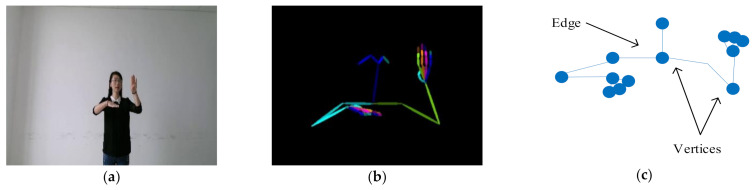
(**a**) The *t*-th RGB frame; (**b**) The skeleton data extracted from RGB frame; (**c**) schematic diagram of graph data.

We first extracted the 2D skeleton data from the RGB frames. The skeleton data can be regard as $X\in {\mathbb{R}}^{T\times V\times C}$, where V is the number of joints in each frame, C is the number of channels, which corresponds to the dimension of each joint, T is the number of frames. As shown in Figure 1, the t-th frame skeleton data is $X_{t}\in R^{V\times C}$. We constructed graph data $G=\left(V,\varepsilon \right)$ from $X_{t}$, where $V=\left(V_{1},\;V_{2}\cdots V_{V}\right)$ is the set of vertices, ε is the set of edges which connect any two vertices in the graph. We set the human joints as the vertices and the bones as the edges to construct the graph from the skeleton.

Because the joint points we extracted are 2-dimensional(2D) coordinates, the intial C is 2. ε represents the connection between V vertices, which can be expressed by the adjacency matrix of $A\subseteq {\mathbb{R}}^{V\times V}$. In addition, A is a symmetric matrix, because the graph we formed is undirected:

(1) $$A_{i,j}=\left\{\begin{array}{c} 1,if\;d\left(V_{i},\;V_{j}\right)=1\\ 0,\;otherwise\;\;\;\;\;\;\;\;\;\;\;\; \end{array}\right.$$

where $d\left(V_{i},V_{j}\right)$ gives the minimum number of human bones between $V_{i}$ and $V_{j}$.

#### 3.1.2. Graph Convolutional Networks

For an action data $X\in {\mathbb{R}}^{T\times V\times C}$, the traditional method [13] alternately used spatial convolution and temporal convolution to extract features. In the spatial convolution, each frame of data $X_{t}\in {\mathbb{R}}^{V\times C}$ is processed separately, which can be described by the following equation:

(2) $$X_{tout}=\sigma \left({\tilde{D}}^{-\frac{1}{2}}\tilde{A}{\tilde{D}}^{-\frac{1}{2}}X_{tin}W\right)\;\tilde{A}=A+I$$

where *A* represented the adjacency matrix of the undirected graph representing intrabody connections, which is defined by (1). I represented the identity matrix and W represented a trainable weight matrix of network. $\tilde{D}$ represented the diagonal degree matrix of $\tilde{A}$, and $\sigma \left(\cdot \right)$ represented a ReLU activation function. Then the input of temporal convolution network (TCN) is $X_{tout}\in {\mathbb{R}}^{T\times V\times C}$, TCN could be designed as a 2D convolutional network: T and V are the convolution scope. We set the kernel as $K_{t}\times 1$, where $K_{t}$ is the number of frames in the accepted field. This means that the temporal convolution operation is only performed in the temporal domain.

### 3.2. Overview

**Figure 2:**
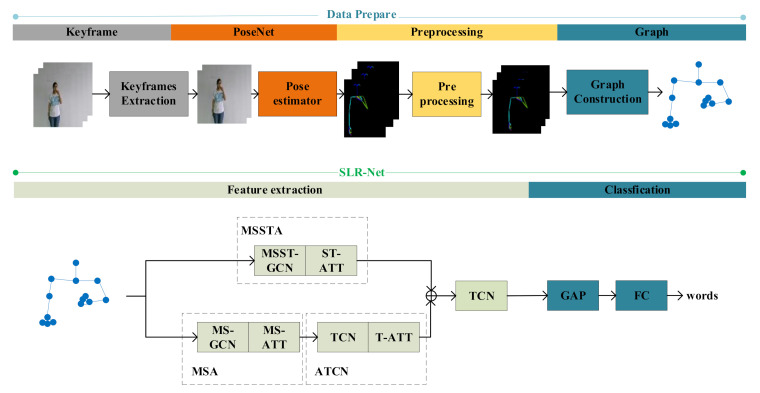
The overview of our proposed method. “⊕” means add operation.

In this section, we will first introduce the overall structure of our proposed method, as shown in Figure 2. Sign language recognition work is divided into two parts: data preparation and sign language recognition network (SLR-Net). The data preparation part can convert the input RGB videos into skeleton data. The keyframes extraction part is optional and suitable for occasions that require high recognition speed. In the part of SLR-Net, we designed a dual-path feature extraction network, the one path uses MSA to extract multi-scale features and then uses ATCN to extract temporal features. The other path uses MSSTA to learn spatiotemporal features directly. Finally, the global average pooling and fully connected layer are used to obtain the classification results.

### 3.3. MSA: Multi-Scale Attention GCN

**Figure 3:**
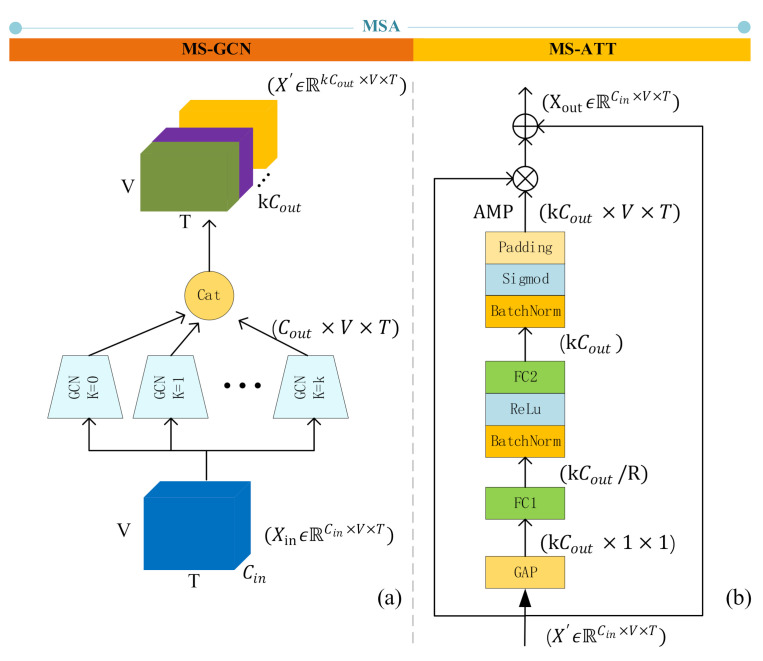
The structure of MSA. (**a**) The structure of MS-GCN, which uses k parallel GCNs to extract features of different levels and concatenate them on the channel layer; (**b**) The structure of MS-ATT, fist perform global average pooling(GAP) on $X^{\prime }$ and turn its dimension into $\left(kC_{\mathrm{out}}\times 1\times 1\right)$. AMP means the attention map, which contains attention information of different scales “⊕” means add operation, “⊗” means matrix dot product operation.

The above GCN algorithm is inefficient in modeling dependencies between remote vertices. In sign language recognition tasks, the distance of joints is usually longer than that of actions, so we proposed multi-scale attention GCN, and named it as MSA. MSA consists of two parts, as shown in Figure 3: one is multi-scale GCN(MS-GCN), which extracts features of different levels; the other is multi-scale attention mechanism (MS-ATT), which assigns attention weights to different scales.

#### 3.3.1. MS-GCN: Multi-Scale GCN

The input of MS-GCN is $X_{in}\epsilon {\mathbb{R}}^{C_{in}\times V\times T}$, which is reshaped by moving *T* into the channel dimension, so that we can use the Equation (3) to perform GCN operations:

(3) $$X^{\prime }=\sigma \left({\tilde{D}}_{\left(k\right)}^{-\frac{1}{2}}{\tilde{A}}_{\left(k\right)}{\tilde{D}}_{\left(k\right)}^{-\frac{1}{2}}X_{in}W_{\left(k\right)}\right)\;{\tilde{A}}_{\left(k\right)}=A_{\left(k\right)}+I$$

where $W_{\left(k\right)}$ is a trainable weight matrix, $A_{\left(k\right)}$ is the k-adjacency matrix:

(4) $$A_{\left(k\right)i,j}=\left\{\begin{array}{c} 1,if\;d\left(v_{i},\;v_{j}\right)=k\\ 0,otherwise\;\;\;\;\;\;\;\;\;\;\;\; \end{array}\right.$$

$A_{\left(k\right)}$ can extend A to more distant neighbors. In particular, $A_{\left(1\right)}=A$. And K controls the number of scales to aggregate. We modify the graph structure by setting a different scale k and perform parallel GCN operations to extract different levels of semantic information. Then concatenate the k outputs of parallel GCNs into $X^{\prime }\epsilon {\mathbb{R}}^{kC_{out}\times V\times T}$. The larger the k is, the easier it is for the GCN to learn the dependencies between remote vertices.

#### 3.3.2. MS-ATT: Multi-Scale Attention Mechanism

**Figure 4:**
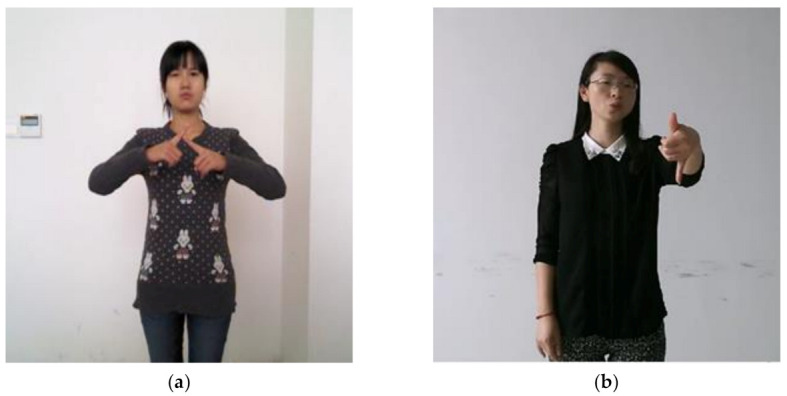
(**a**) Chinese sign language word “person”; (**b**) Chinese sign language word “go out”.

In the MS-GCN, graphs of different scales can learn features of different levels, which alleviates the problem that ordinary GCN is difficult to model the relationship between remote vertices. However, MS-GCN simply stacks the extracted features together. In the isolate sign language recognition task, some word needs to model the long-distance vertices relationship. For example, the “person” in Chinese sign language is expressed by the touch of the left and right fingers. There are also some words that pay more attention to the closer vertices. For instance, the Chinese sign language “go out” is expressed by the left thumb and the little finger (Figure 4). Therefore, we proposed MS-ATT, which gives the network the ability to weight different scale features according to different sign language words.

In the multi-scale aggregation scheme, graphs of different scales can learn features of different distance levels, which alleviates the problem that ordinary GCN is difficult to model the relationship between remote vertices. However, in sign language vocabulary recognition, different vocabulary needs different scale graphs. Therefore, we proposed MS-ATT, which gives the network the ability to weight different features according to different sign language vocabularies.

The input of MS-ATT is $X^{\prime }\epsilon {\mathbb{R}}^{kC_{out}\times V\times T}$, which is the output of MS-GCN. First, $X^{\prime }$ is averaged in spatial dimension V and temporal dimension T, get $f\epsilon {\mathbb{R}}^{K\times C_{out}\times 1\times 1}$. Then add FC1 fully connection layer to extract the attention information about the multi-scale semantic features, the number of neurons in this layer is $K\times C_{out}/R,\left(R>1\right)$. And then use the FC2 fully connection layer to restore the features to the original dimension of $K\times C_{out}$, and then copy it on the dimensions V and T to get the attention map (AMP). Attention map and $X^{\prime }$ are dotted to add attention information to the feature. Among them, R is an adjustable parameter, and we empirically set it to 2. The multi-scale attention mechanism can be described by the following equation:

(5) $$X_{\mathrm{out}}=X^{\prime }+X^{\prime }⊗\mathrm{AMP}$$

where “⊗” means matrix dot product operation.

### 3.4. ATCN: Attention Enhanced Temporal Convolutional Network

**Figure 5:**
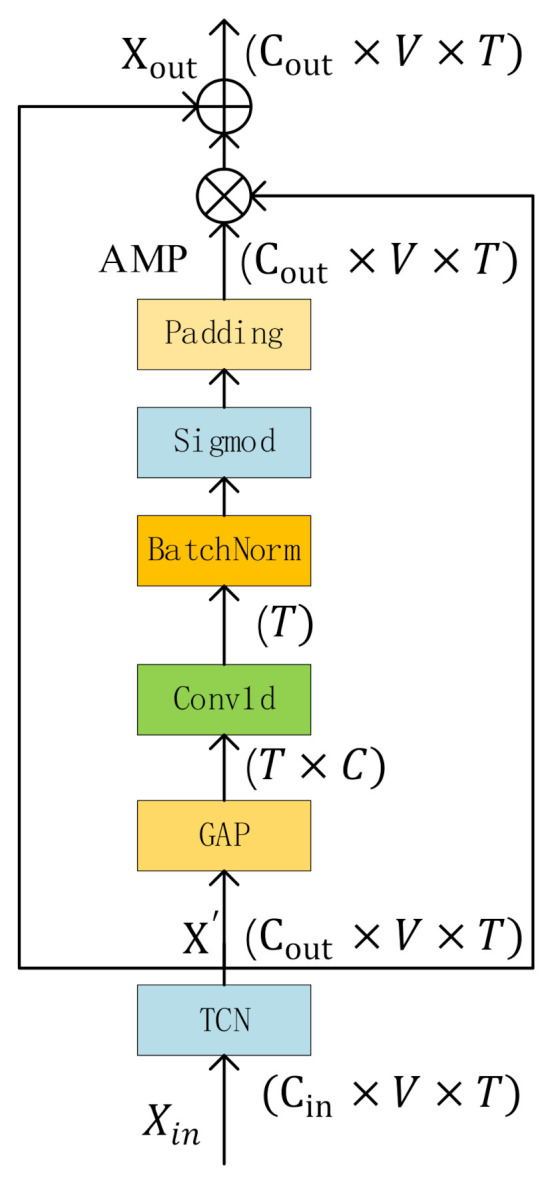
The structure of MSA. AMP means the attention map, which contains attention information of different time.

Sign language actions are usually composed of several different key actions and their transitions. Therefore, sign language lasts a long time and have different importance at different times. Frames containing more discriminative information should get more attention. Therefore, we design attention enhanced temporal convolution network (ATCN), which can pay more attention to the key actions in the sign language rather than the transition actions between key actions (Figure 5).

After MSA extracts multi-scale semantic features, we use the ATCN network to further extract temporal features. It is composed of a temporal convolutional network (TCN) and a temporal attention mechanism (T-ATT). TCN follows the design of [14]. We send the TCN’s output $X^{\prime }$ to the T-ATT. We first perform a global average pooling operation to average the V dimension, and get a feature matrix with dimension of C×T, moving T to the batch dimension, and then perform a 1D convolution, the dimension of output is T×1, after the sigmoid function is activated, copy it in the V dimension and the $C_{out}$ dimension to get the attention map. The dimension of attention map is $C_{out}\times V\times T$. $X_{out}$ is calculated as in Equation (5).

### 3.5. MSSTA: Multi-Scale Spatiotemporal Attention Network

**Figure 6:**
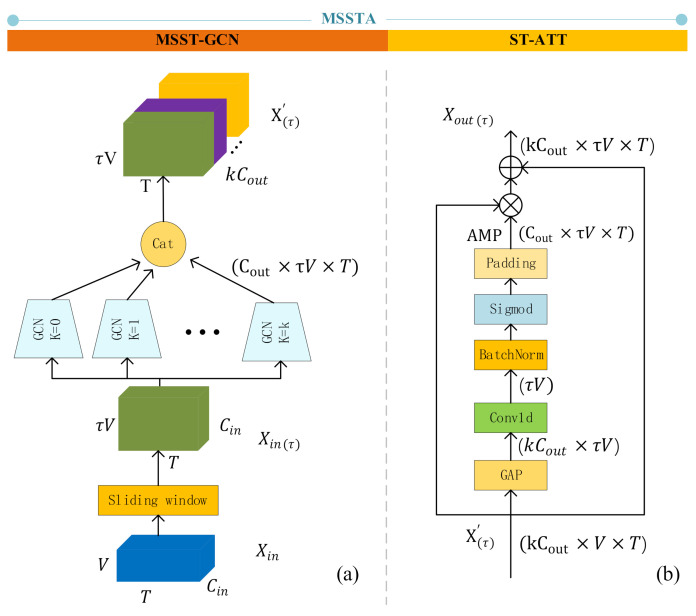
The structure of MSSTA. (**a**) The structure of MSST-GCN, which uses k parallel GCNs to extract features of different levels and concatenate them on the channel layer; (**b**) The structure of ST-ATT, GAP means global average pooling, which turns the dimension of $X_{out\left(\tau \right)}$ into $kC_{\mathrm{out}}\times \tau V$. AMP means the attention map.

Many existing methods divide spatiotemporal features into temporal features and spatial features to extract separately, which separates the inherent connection of time and space. To solve this problem, we proposed a multi-scale spatiotemporal attention network (MSSTA) to learn spatiotemporal features directly (Figure 6).

#### 3.5.1. MSST-GCN: Multi-Scale Spatiotemporal GCN

First, we set a time sliding window, a window includes τ frames, which could be viewed as a spatiotemporal subgraph $G_{\left(\tau \right)}=\left(V_{\left(\tau \right)},\;\varepsilon_{\left(\tau \right)}\right)$, where $V_{\left(\tau \right)}=\left(V_{1},\;V_{2}\cdots V_{\tau V}\right)$. $G_{\left(\tau \right)}$ is the union of all vertices sets across τ frames in the window. The edge set $\varepsilon_{\left(\tau \right)}$ is defined by tiling $\tilde{A}$ into a block adjacency matrix ${\tilde{A}}_{\left(\tau \right)}$ (Equation (6)):

(6) $${\tilde{A}}_{\left(\tau \right)}=\left[\begin{array}{ccc} \tilde{A} & \cdots & \tilde{A}\\ \vdots & \ddots & \vdots \\ \tilde{A} & \cdots & \tilde{A} \end{array}\right]\epsilon R^{\tau V\times \tau V}$$

This means that the *j*-th vertex of the i-th vertex subset $V_{i}$ will be connected to the adjacent vertices in the one-hop neighbor in $V_{i}$, and will also be connected to all the j-th vertices and its one-hop neighboring nodes in the other τ−1 vertices subsets. Then the input become $X_{in\left(\tau \right)}\in {\mathbb{R}}^{T\times \tau V\times d}$. We can get the multi-scale spatiotemporal GCN as Equation (7):

(7) $$X_{\left(\tau \right)}^{\prime }=\sigma \left({\tilde{D}}_{\left(\tau ,k\right)}^{-\frac{1}{2}}{\tilde{A}}_{\left(\tau ,k\right)}{\tilde{D}}_{\left(\tau ,k\right)}^{-\frac{1}{2}}X_{\left(\tau \right)}W_{\left(k\right)}\right)\;{\tilde{A}}_{\left(\tau ,k\right)}=\left[\begin{array}{ccc} {\tilde{A}}_{\left(k\right)} & \cdots & {\tilde{A}}_{\left(k\right)}\\ \vdots & \ddots & \vdots \\ {\tilde{A}}_{\left(k\right)} & \cdots & {\tilde{A}}_{\left(k\right)} \end{array}\right]\epsilon R^{\tau V\times \tau V}$$

where ${\tilde{D}}_{\left(\tau ,k\right)}$ is the diagonal degree matrix of ${\tilde{A}}_{\left(\tau ,k\right)}$.

#### 3.5.2. ST-ATT: Spatiotemporal Attention Mechanism

The MSST-ATT network can directly learn spatiotemporal features. However, in sign language recognition, the importance of joints varies for different actions and times; and some hand joints output by PoseNet are inaccurate because of the finger occlusion problem. These inaccurate joints are often of little significance for understanding the semantics of words. Therefore, we combined multi-scale GCN to propose a multi-scale 3D spatiotemporal attention mechanism, which allows the algorithm to pay more attention to important joint points and reduces the influence of inaccurate hand joint points on the final word recognition result.

### 3.6. Two-Stream SLR-Net

**Figure 7:**
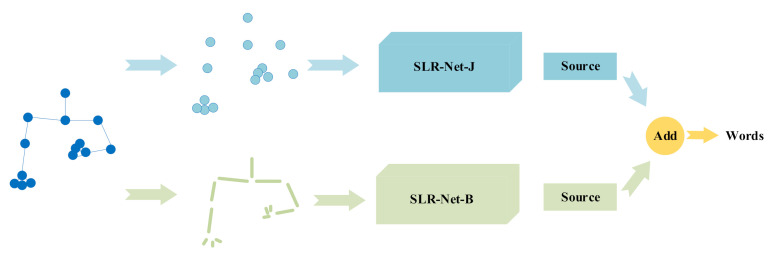
The structure of two-stream fused SLR-Net (SLR-Net-J+B).

Inspired by the two-stream network [14], we applied the same strategy to SLR-Net, as shown in Figure 7. Specifically, the input of the first-stream is the joints data, and the input of the other-stream is the bones data between the joints. The scores output by the two-stream networks is added as a new score to recognize sign language words. The joints data is extracted by PoseNet, and the bone data can be calculated from the joint point data. For example, one joint coordinates are $V_{i}=\left(x_{i},y_{i}\right)$, another joint coordinates are $V_{j}$ = $\left(x_{j},y_{j}\right)$, if there is a human bone between $V_{i}$ and $V_{j}$, then the bone data can be expressed as $B_{i,j}=\left(x_{j}-x_{i},y_{j}-y_{i}\right)$. Bones data can be regarded as high-level information of joints data. Joints data expresses position information clearly, while bone data pays more attention to length and direction information. Both of them have important roles in understanding sign language. Therefore, a two-stream fused network will effectively improve the recognition accuracy.

### 3.7. Keyframes Extraction

A sign language action consists of many frames, these frames can be divided into keyframes and transition frames, where keyframes include specific gestures and regular actions. If the keyframes can be extracted accurately, it will help to recognize sign language efficiently. Video keyframes extraction methods include: Perceived Motion Energy Model [43], Visual frame Descriptors [44], Motion Attention Model [45], Multiple Visual Descriptor Features [46], Motion focusing [47], Camera Motion and Object Motion [48], Visual Attention Clues [49].

**Figure 8:**
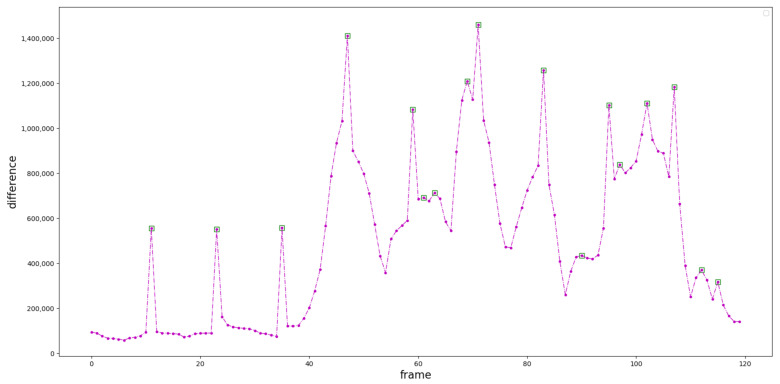
The output of Inter-frame difference method. The purple dots are the differences between two adjacent frames, and the green rectangle boxes are local extremes. The horizontal axis is the number of frames, and the vertical axis is the difference value between frames.

We designed a keyframes extraction algorithm based on image entropy. First, we divided the video into n frames and grouped them into n categories, and used the inter-frame difference method [50] to measure the similarity to obtain n−1 difference values as shown in Figure 8. Then we find out the k−1 local extremes of the difference sequence, and use these local extremes to aggregate n classes into k classes (k<n), the video frames in the same class are similar, and the frames between classes are not similar. If the definition of a picture in the class is greater than the threshold, select the frame with the largest image entropy in the class as keyframe, otherwise, the picture in this class is considered to be too fuzzy and unrepresentative, and select the keyframe from the next class.

In order to verify the rationality of using local maximum to segment the videos, we used paired T test to analyze if the two groups were significantly different. The difference values of local maximum were set as group 1 (green rectangle boxes in Figure 8), and that of the others were set as group 2 (the other purple dots in Figure 8). The statistical results were shown in Table 1, which indicated that the average score of group 1 was significantly higher than that of the group 2.

In order to avoid the problem of inaccurate recognition of joint points caused by blurred frames, the frames whose definition is less than the threshold should be removed. Considering that the definition of the image can be judged by its edge, and the gradient of the image can well reflect the edge gray of the target object in the image (Equation (8)). The definition of the image definition based on the Tenengrad gradient function is as follows:

(8) $$D\left(f\right)=\Sigma_{y}\Sigma_{x}\left|G\left(x,y\right)\right|$$

where $G\left(x,y\right)$ is the convolution of the Laplacian operator at the pixel (*x*,*y*), and the Laplacian operator is:

(9) $$L=\frac{1}{6}\left[\begin{array}{ccc} 1 & 4 & 1\\ 4 & -20 & 4\\ 1 & 4 & 1 \end{array}\right]$$

The concept of information entropy was proposed by Shannon to measure the uncertainty of information [51]. An image is a two-dimensional discrete signal and the amount of image information can be measured by information entropy, which can also be called image entropy. For a gray image I with a gray level of L (1<L<256)  and size of  M×N, use f(x, y) to represent the grayscale of the pixels in the image with coordinates (x, y) Value, the range of f(x, y) is [0, *L* − 1]. Let $f_{i}$ be the number of gray levels i in the image, the probability of gray level i is:

(10) $$p_{i}=\frac{f_{i}}{M\times N}\;,i=\left(0,1,\ldots ,L-1\right)\;$$

**Figure 9:**
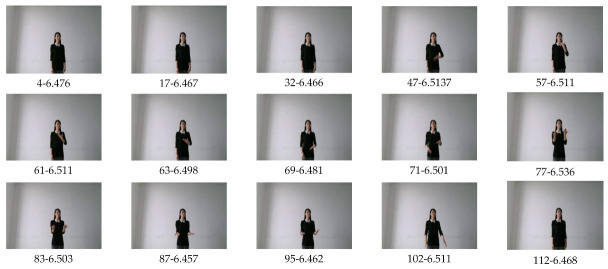
Keyframes of Chinese Sign Language word “Heavy”. Below the picture are the frame number and the values of image entropy. For example, 4-6.476 means the frame number is 4, and the image entropy is 6.476.

The image entropy is:

(11) $$H=-\overset{L-1}{\underset{i=0}{\sum }}P_{i}\log\left(P_{i}\right)$$

where $P_{i}\in \left(0,1\right)$,$\overset{L-1}{\underset{i=0}{\sum }}P_{i}=1$; log represents a logarithm, and the base of derivation in information theory is 2. After filtering out the blur frames with image definition, the frame with the largest image entropy is selected as keyframe, and the final selected keyframes are as follows (Figure 9):

The algorithm in this paper needn’t set the cluster number, and the algorithm will automatically find keyframes. Using the keyframe extraction algorithm on CLS-500 dataset, we can reduce the number of frames from a maximum of 220 to a maximum of 40, which greatly decreases the amount of calculation. At the same time, the keyframe extraction could be regarded as a re-sampling work, which alleviates the influence of different people’s inconsistent movement speed and blurred frames.

### 3.8. Skeleton Data Normalization

**Figure 10:**
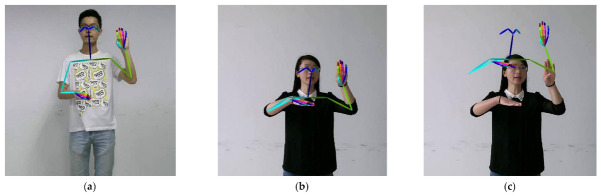
Skeleton data normalization example. (**a**) Benchmark sign language speaker; (**b**) Joints data before normalization; (**c**) Joints data after normalization.

In sign language recognition, the motion trajectories of different sign language speakers often vary due to the differences in height and body shape, which harms sign language recognition. To solve this problem, we proposed a normalization algorithm: firstly, selected a benchmark sign language speaker, then translated and zoomed the joints data of other sign speakers until their neck joint position and shoulder width were the same as the benchmark sign language speaker (Figure 10).

## 4. Experiments

### 4.1. Datasets

#### 4.1.1. CSL-500

The Chinese Sign Language Dataset (CSL-500) contains 25,000 labeled video samples, taken by 50 operators, which has multiple modal data, including RGB, depth, and skeleton data. There are 500 words in the dataset, which contains 50 examples of each word and 21 body joints coordinate sequences. Each video instance is marked by a professional Chinese sign language teacher. The specific CSL500 dataset parameters are shown in Table 2.

#### 4.1.2. DEVISIGN-L 

**Figure 11:**
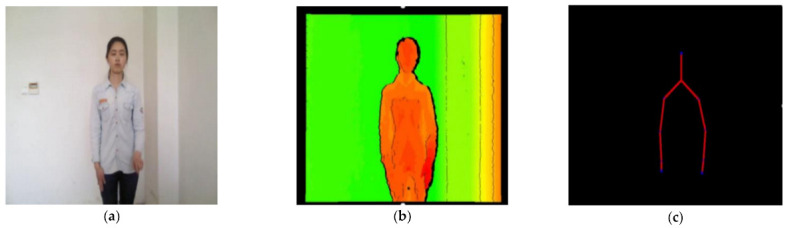
Example of DEVISIGN-L dataset. (**a**) RGB data; (**b**) depth image data; (**c**) Skeleton data.

The DEVISIGN-L dataset contains 2000 Chinese sign language words, which is currently the largest Chinese sign language dataset with the largest vocabulary (Figure 11). The data was recorded by eight sign language speakers. For four of the speakers, the data of all vocabularies were recorded twice; for the other four speakers, the data was recorded only once. The following table summarizes the details of DEVISIGN-L. The details of DEVISIGN-L are shown in Table 3.

### 4.2. Evaluation Metrics

**Figure 12:**
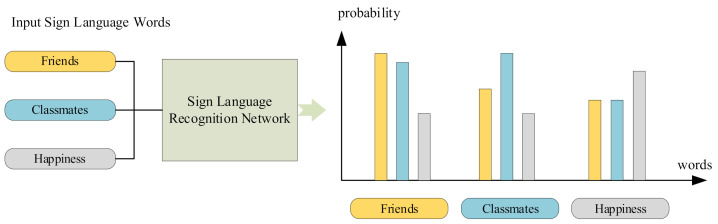
The output of each sample is a probability vector.

Sign language vocabulary recognition can be regarded as a multi-classification task. In such tasks, the artificial neural network will output a probability vector. The dimension of the vector is the same as the number of categories, indicating the probability of classifying the sample into each category (Figure 12).

This work used top-1 accuracy and top-5 accuracy to evaluate the performance of the algorithm. Top-1 accuracy: Input a sign language word, and if the word with the highest probability output by the algorithm is consistent with the ground-truth, the word is considered to be correctly recognized. Top-5 accuracy: Input a sign language word, and if the top five words with the highest probability output by the algorithm contain the ground-truth, the word recognition is correct. The calculation formula for accuracy is as follows (Equation (12)):

(12) $$accuracy=\frac{Number\;of\;correctly\;recognized\;samples}{The\;total\;number\;of\;samples\;in\;the\;test\;set}$$

### 4.3. Implementation Details

Although both of these two datasets provide skeleton data generated by the Kinect device, the joints data is only body parts, and the joint data of the hands are missing, which cannot be used for sign language recognition. This work used the RGB data in the CSL-500 and DEVISIGN-L datasets to generate 2D skeleton data of hands, body, and part of the face, and then used the GCN algorithm for sign language recognition. All experiments are based on the PyTorch deep learning framework [52].

Each vocabulary of the CSL-500 dataset has fifty corresponding sign language videos. We randomly divide 90% of the data into the training set and use the remaining 10% as the test set. The dataset has a total of 25,000 videos, and we resized the number of frames of each video to 220. From each frame, we extracted 52 human joints, and there are 51 human bones between them. In the model of SLR-Net-J, the number of vertices is 52 and the number of edges is 51. And in the model of SLR-Net-B, the number of vertices and edges are both 51. There are three bones data and four joints data for each finger. A total of 50 epochs were trained, the batch size was 12. The initial learning rate is set to 0.1, attenuates by a factor of 10 at the 30th and 40th epoch. When using the keyframe algorithm, we set the maximum number of frames to 40, set the batch size to 80, and leave the remaining parameters unchanged. The entire network was trained end-to-end using Stochastic Gradient Descent (SGD) with a momentum of 0.9.

For the DEVISIGN-L dataset, its vocabulary size is 2000, but the sample size of each vocabulary is only 12. We use 75% of the data as the training set and 25% of the data as the test set. Three sign speakers were randomly selected from the four sign speakers who were only collected once, and the data of these three presenters were set as the test set. The dataset has a total of 24,000 videos, and we resized the number of frames of each video to 260. The other experimental details were the same as the CSL-500 dataset.

### 4.4. Ablation Experiment

To verify the effectiveness of the various modules we proposed, we conducted a large number of experiments on the two Chinese sign language datasets: CSL-500 and DEVISIGN-L. We used ST-GCN [13] as our baseline, which was originally used for action recognition, and we modified it to test the sign language dataset.

#### 4.4.1. Skeleton Data Normalization

We used the baseline algorithm to test the normalization algorithm on the CSL-500 dataset, and the experimental results were shown in Table 4:

The experimental results showed the effectiveness of the normalization algorithm. In the following experiments, we used the normalized skeleton data for experiments.

#### 4.4.2. Dual-Path Feature Extraction Network

In the feature extraction part, we designed a dual-path network fusion structure, one of which was MSSTA, and the other was MSA + ATCN. From Table 5 we can see that the accuracy of MSSTA is slightly higher than MSA + ATCN, because MSSTA could directly learn spatiotemporal features. Finally, they were compared with the complete SLR-Net, and the experiment proved that the dual-path fusion could effectively improve the recognition accuracy.

#### 4.4.3. Attention Mechanism

In this section, we verified the validity of MS-ATT, ST-ATT, and T-ATT, respectively. As shown in Table 6, SLR-Net added the above three attention mechanisms, SLR-Net (No-ATT) did not add any attention mechanism.

From Table 6, even without any attention mechanism, the recognition accuracy of SLR-Net (No-ATT) was still better than the baseline. This is because SLR-Net can learn farther spatial dependence and has the ability to directly learn spatiotemporal features, which is more suitable for sign language recognition. Compared with SLR-Net (No-ATT), adding MS-ATT, ST-ATT, and T-ALL can also increase the accuracy by 0.74%, 0.78% and 0.46% respectively. Among them, ST-ATT performed best. SLR-Net was the complete model with three attention mechanisms, whose accuracy was 2.96% higher than that of the baseline.

The attention mechanisms have also been experimented on the DEVISIGN-L dataset. As shown in Table 7. This dataset is more challenging because the dataset has a large vocabulary, but the number of samples per vocabulary is only 12, the accuracy of baseline recognition on this dataset was only 44.6%. The SLR-Net and three attention mechanisms have significantly improved recognition accuracy. Compared with the SLR-Net without attention (No-ATT), the addition of MSATT increased the accuracy by 3.62%, ST-ATT increased the accuracy by 4.7%, and T-ATT increased the accuracy by 5.1%. Adding three attention mechanisms at the same time, SLR-Net increased by 5.95%.

#### 4.4.4. Two-Stream Framework

Here we verified the effectiveness of the two-stream framework. In Table 8, SLR-Net-J means that the input of the network is joints data, SLR-Net-B means that the input is bone data, and SLR-Net-J+B means the fusion of the two-stream. It can be seen that although the recognition accuracy of SLR-Net-B was 0.68% lower than that of SLR-Net-J, the two-stream fusion has reached the optimal accuracy rate of 98.08%.

#### 4.4.5. Keyframes Extraction

We used the keyframe extraction algorithm to extract a subset of CSL-500 dataset: CSL-500-key. The experimental results on the CSL-500-key dataset were as Table 9:

It can be seen that the network and attention mechanism we proposed also perform well on the CSL-500-key dataset. Compared with the baseline, the method in this paper increases the accuracy by 16.64%. The running time is compared in Table 10. Based on the analysis of Table 10, the keyframe algorithm will sacrifice 3.84% accuracy, but it saves 46.3% of the time, which greatly improves the recognition efficiency.

### 4.5. Comparison to Other State-of-the-Art Methods

We compared our method with other methods; the recognition accuracy compared with other methods on CSL-500 is shown in Table 11; the recognition accuracy compared with other methods on DEVISIGN-L is shown in Table 12. The Tsn, I3d, Tsm and Attention 3D-CNN are CNN based methods, the B3D-ResNet is based on CNN and RNN. The above methods was designed for sign language recognition. The ST-GCN, 2S-AGCN are GCN based method for action recognition, we modified them to recognize sign language words to compare with SLR-Net.

Table 11 and Table 12 showed that in sign language recognition tasks, GCN-based algorithms outperformed the other CNN and RNN based sign language recognition algorithms and our proposed SLR-Net worked well on the two public datasets.

## 5. Conclusions

This paper proposed a new GCN based sign language vocabulary recognition network: SLR-Net. The article introduced a series of processes from data preparation to vocabulary recognition, providing new ideas for sign language recognition. SLR-Net is composed of three sub-modules MSSTA, MSA, and ATCN. They can extract features between vertices at long distances and have the ability to directly learn spatiotemporal features. The ablation experiment also verified our original hypothesis. We have also added three different attention mechanisms to each sub-module to further improve the robustness of the model. Besides, a keyframe extraction algorithm is proposed, which can greatly improve efficiency by sacrificing a little accuracy. Finally, we did a lot of experiments on two large-scale sign language datasets and reached the best performance on both of them.

## Figures and Tables

**Table 1:** Paired T test. (Std. is an abbreviation of Standard. Df means degree of freedom. Sig. is an abbreviation of Significant.).

	Paired Differences					t	df	Sig. (2-Tailed)
	Mean	Std. Deviation	Std. Error Mean	95% Confidence Interval of the Difference				
				Lower	Upper			
sample 1–sample 2	304,990.5000	192,013.19871	51,317.68595	194,125.37975	415,855.62025	5.943	13	0.000

**Table 2:** Details of the CSL-500 dataset.

RGB Resolution	1280 × 720
Depth Resolution	512 × 424
Number of joints	21 (only body)
Fps	30
Per video duration(s)	2–5
Number of videos per word	50
Vocabulary	500
Total videos of samples	25,000

**Table 3:** Details of the DEVISIGN-L dataset.

RGB Resolution	640 × 480
Number of joints	21 (only body)
Fps	30
Per video duration(s)	2–5
Number of videos per word	12
Vocabulary	2000
Total videos of samples	24,000

**Table 4:** Action recognition accuracies by baseline, tested on CSL-500 dataset.

Method	Top-1 Accuracy (%)	Top-5 Accuracy (%)
No normalize	93.8	99.56
Normalized	94.4	99.64

**Table 5:** The experiment of dual-path feature extraction network, MSSTA is the one path of SLR-Net and MSA + ATCN is another path. Tested on CSL-500 dataset.

Method	Top-1 Accuracy (%)	Top-5 Accuracy (%)
MSSTA	96.44	99.64
MSA + ATCN	95.68	99.68
SLR-Net	97.36	99.68

**Table 6:** Sign languages recognition accuracies by SLR-Net with attention mechanisms, tested on CSL-500 dataset.

Method	Top-1 Accuracy (%)	Top-5 Accuracy (%)
ST-GCN [13]	94.4	99.64
SLR-Net (No-ATT)	96.1	99.6
SLR-Net (only MS-ATT)	96.84	99.64
SLR-Net (only ST-ATT)	96.88	99.72
SLR-Net (only T-ATT)	96.56	99.68
SLR-Net	97.36	99.68

**Table 7:** Sign languages recognition accuracies by SLR-Net with attention mechanisms, tested on DEVISIGN-L dataset.

Method	Top-1 Accuracy (%)	Top-5 Accuracy (%)
ST-GCN [13]	44.6	69.68
SLR-Net (No-ATT)	59.62	81.28
SLR-Net (only MS-ATT)	62.88	81.73
SLR-Net (only ST-ATT)	64.32	84.17
SLR-Net (only T-ATT)	64.72	83.53
SLR-Net	65.57	84.27

**Table 8:** The results of two-stream framework on CSL-500 dataset.

Method	Top-1 Accuracy (%)	Top-5 Accuracy (%)
SLR-Net-J	97.36	99.68
SLR-Net-B	96.68	99.72
SLR-Net-J+B	98.08	99.84

**Table 9:** Sign languages recognition accuracies by SLR-Net with attention mechanisms, tested on CSL-500-key dataset.

Method	Top-1 Accuracy (%)	Top-5 Accuracy (%)
ST-GCN [13]	76.4	95.32
2S-AGCN [14]	87.48	97.8
SLR-Net (No-ATT)	89.08	97.6
SLR-Net (only MS-ATT)	92.6	98.92%
SLR-Net (only ST-ATT)	92.88	98.8%
SLR-Net (only T-ATT)	91.64	99.00%
SLR-Net	93.04	98.92%

**Table 10:** The accuracy and runtime comparisons.

Method	Runtime per Sample	Top-1 Accuracy (%)
SLR-Net	19 ms	96.88
SLR-Net + keyframe	8.8 ms	93.04

**Table 11:** The accuracy comparisons with state-of-the-art methods on the CSL-500 dataset.

Method	Year	Top-1 Accuracy (%)	Top-5 Accuracy (%)
Tsn [53]	2016	74.96	91.00
I3d [54]	2017	89	98.16
Tsm [55]	2019	90.84	99.16
Attention 3D-CNN [6]	2018	88.70	-
B3D-ResNet [35]	2019	86.9	-
ST-GCN [13]	2018	94.40	99.64
2s-AGCN-J [14]	2019	95.6	98.56
2s-AGCN-B [14]	2019	95.84	98.52
2s-AGCN-J+B [14]	2019	96.72	99.72
SLR-Net-J (ours)	-	97.36	99.68
SLR-Net-B (ours)	-	96.68	99.72
SLR-Net-J+B (ours)	-	98.08	99.84

**Table 12:** The accuracy comparisons with state-of-the-art methods on the DEVISIGN-L dataset.

Method	Year	Top-1 Accuracy (%)	Top-5 Accuracy (%)
Tsn [53]	2016	2.13	5.87
I3d [54]	2017	5.98	17.32
Tsm [55]	2019	25.4	51.63
ST-GCN [13]	2018	44.60	69.68
2S-AGCN [14]	2019	62.68	82.40
SLR-Net (ours)	-	65.57	84.27

## Data Availability

Restrictions apply to the availability of these datasets. CSL-500 dataset was obtained from University of Science and Technology of China and are available from (http://home.ustc.edu.cn/~pjh/openresources/cslr-dataset-2015/index.html) with the permission of University of Science and Technology of China. DEVISIGN-L dataset was obtained from Institute of Computing Technology, Chinese Academy of Sciences and are available from (http://vipl.ict.ac.cn/homepage/ksl/data.html#database) with the permission of Institute of Computing Technology, Chinese Academy of Sciences.
